# *Staphylococcus aureus* exhibits heterogeneous siderophore production within the vertebrate host

**DOI:** 10.1073/pnas.1913991116

**Published:** 2019-10-14

**Authors:** William J. Perry, Jeffrey M. Spraggins, Jessica R. Sheldon, Caroline M. Grunenwald, David E. Heinrichs, James E. Cassat, Eric P. Skaar, Richard M. Caprioli

**Affiliations:** ^a^Mass Spectrometry Research Center, Vanderbilt University, Nashville, TN 37232;; ^b^Department of Chemistry, Vanderbilt University, Nashville, TN 37232;; ^c^Vanderbilt Institute for Infection, Immunology, and Inflammation, Vanderbilt University, Nashville, TN 37232;; ^d^Department of Biochemistry, Vanderbilt University, Nashville, TN 37232;; ^e^Department of Pathology, Microbiology, and Immunology, Vanderbilt University Medical Center, Nashville, TN 37232;; ^f^Department of Microbiology and Immunology, University of Western Ontario, London, ON N6A 3K7, Canada;; ^g^Department of Pediatric Infectious Diseases, Vanderbilt University Medical Center, Nashville, TN 37232;; ^h^Department of Biomedical Engineering, Vanderbilt University, Nashville, TN 37232;; ^i^Department of Pharmacology, Vanderbilt University, Nashville, TN 37232;; ^j^Department of Medicine, Vanderbilt University, Nashville, TN 37232

**Keywords:** siderophore, metallophore, mulitmodal molecular imaging, infectious disease, nutritional immunity

## Abstract

Siderophores, iron-scavenging small molecules, are fundamental to bacterial nutrient metal acquisition and enable pathogens to overcome challenges imposed by nutritional immunity. Multimodal imaging mass spectrometry allows visualization of host−pathogen iron competition, by mapping siderophores within infected tissue. We have observed heterogeneous distributions of *Staphylococcus aureus* siderophores across infectious foci, challenging the paradigm that the vertebrate host is a uniformly iron-depleted environment to invading microbes.

Metals are required by organisms to carry out metabolic processes (1). During infection, host metalloproteins sequester nutrient metals to prevent microbial colonization, a process termed nutritional immunity (2, 3). Bacteria have evolved sophisticated metal acquisition strategies, including the use of siderophores (4, 5). Siderophores are secondary metabolites (<1 kDa) characterized by a high binding affinity for iron (Fe) (dissociation constant [*K*_*d*_] > 10^−30^ M) (5, 6). *Staphylococcus aureus* is an opportunistic pathogen that utilizes siderophores for Fe acquisition, and siderophore production is required for maximum virulence (5, 7).

One hallmark of *S. aureus* infection is the formation of tissue abscesses (8). Abscess architecture consists of staphylococcal abscess communities (SACs) segregated from host tissue by layers of necrotic and healthy innate immune cells (Fig. 1*A*) (9). In Fe-limiting environments, transcriptional repression of the *S. aureus* ferric uptake regulator (Fur) regulon is ceased, and bacteria increase expression of Fe acquisition machinery (5). Known mechanisms of staphylococcal Fe acquisition include heme uptake, inorganic Fe transport, and secretion of the siderophores staphyloferrin A (SA) and staphyloferrin B (SB) (10, 11). Emerging literature suggests that abscesses exhibit molecular heterogeneity, and, therefore, SACs elaborate differential gene expression, questioning the spatial and temporal importance of siderophores (12). However, the distribution of bacterial siderophores within vertebrate tissue has not been visualized. We sought to revisit the paradigm that bacteria are uniformly Fe-starved during vertebrate colonization, by mapping siderophore distributions in infected tissues using high-performance matrix-assisted laser desorption/ionization Fourier transform ion cyclotron resonance imaging mass spectrometry (MALDI FT-ICR IMS) (2, 5, 12–19).

**Fig. 1.**
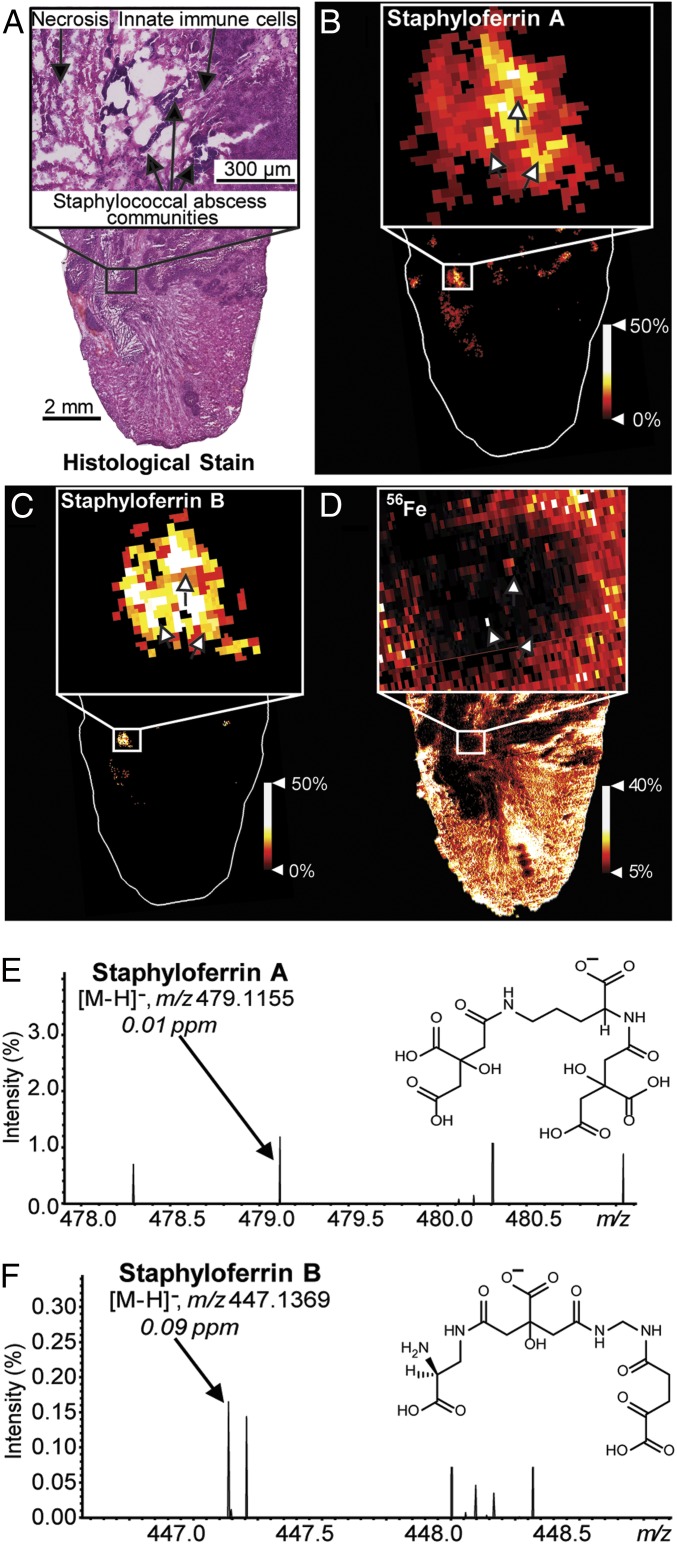
MALDI IMS reveals siderophores SA and SB within the infectious environment. (*A*) H&E stained abscesses. A zoom shows abscess morphology containing SACs (arrows). (*B*) MALDI FT-ICR IMS reveals SA colocalizing with infection sites. (*C*) SB is more closely localized to SACs than SA. (*D*) Fe distributions colocalize with select SACs (arrows). (*E*) A spectral zoom shows a signal corresponding to SA at *m/z* 479.1155, 0.01 ppm mass error, and the chemical structure of SA, [M-H]^−^. (*F*) A spectral zoom shows a signal corresponding to SB at *m/z* 447.1369, 0.09 ppm mass error, and the chemical structure of SB, [M-H]^−^.

Mice were infected with wild-type *S. aureus*, and tissue was harvested 7 d post infection (DPI) and frozen on dry ice (20). Tissues were sectioned serially for hematoxylin/eosin (H&E) staining, MALDI IMS, and ^56^Fe analysis using laser ablation—inductively coupled plasma (LA-ICP) IMS (Fig. 1 *A–D*). Using MALDI IMS, ions corresponding to *S. aureus* siderophores SA [M-H]^−^ at *m*/*z* 479.1155 (mass accuracy: 0.01 parts per million [ppm] error) and SB [M-H]^−^ at *m*/*z* 447.1369 (mass accuracy: 0.09 ppm error) were observed within tissue (Fig. 1 *B* and *C*). Tentative molecular identifications are based on accurate mass measurements. The SA molecular assignment was validated by MALDI IMS of mice infected with a *S. aureus* mutant genetically inactivated for SA production (∆*sfa*) (21). Methods, supplemental information, and raw data can be found at https://doi.org/10.6084/m9.figshare.9617633.v4.

Both siderophores localize to infection sites and expand beyond the perimeter of the SACs, highlighting the metabolic effort of *S. aureus* to acquire Fe (Fig. 1 *A–C*). Differences in siderophore production can be observed across abscesses. Comparing the 2 siderophore distributions, SA has increased prevalence at most infection sites. However, some foci show higher relative abundances of SB, suggesting differential Fe starvation at these sites. Notably, little to no siderophore is detected within some abscesses. Attempts to detect ferric SA and ferric SB were unsuccessful. It is possible that the complex is not stable due to the high basicity of the MALDI matrix or does not survive the MALDI process. Alternatively, but less likely, ferric SA and ferric SB are present but at low abundance and not detectable. Fe is largely excluded from infection sites (Fig. 1*D*). However, some pixels show colocalization of Fe to SACs (Fig. 1*D*, arrows), presumably highlighting successful acquisition of the metal.

To further investigate host−pathogen Fe competition, heart, liver, and kidney lesions were compared using a multimodal approach integrating IMS, H&E staining, and fluorescence microscopy. Mice were infected with *S. aureus P*_*isdA*_*gfp*, where green fluorescent protein (GFP) expression is driven by the Fur-regulated *isdA* promoter (12). After 10 DPI, tissues presented with abscesses (Fig. 2). Fluorescence micrographs of GFP expression allow for a MALDI IMS-compatible technique to visualize SAC responses to host Fe sequestration (Fig. 2). Fig. 2 shows siderophores colocalizing with GFP expression. Comparing H&E stains to Fe distributions, some SACs colocalize with Fe, while others do not. This observation supports nutritional heterogeneity of SACs and suggests differential molecular responses. Siderophores are produced by *S. aureus* across necrotic abscesses in all tissues examined. In the heart abscess, SA extends beyond the abscess, highlighting SA diffusion (Fig. 2*B*). Siderophore distributions localize to GFP expression. Fe distributions within the liver and kidney lesions (Fig. 2 *A* and *C*) localize to GFP absence and lessened siderophore signals. These data highlight differential siderophore production across abscesses from liver, heart, and kidney tissues.

**Fig. 2.**
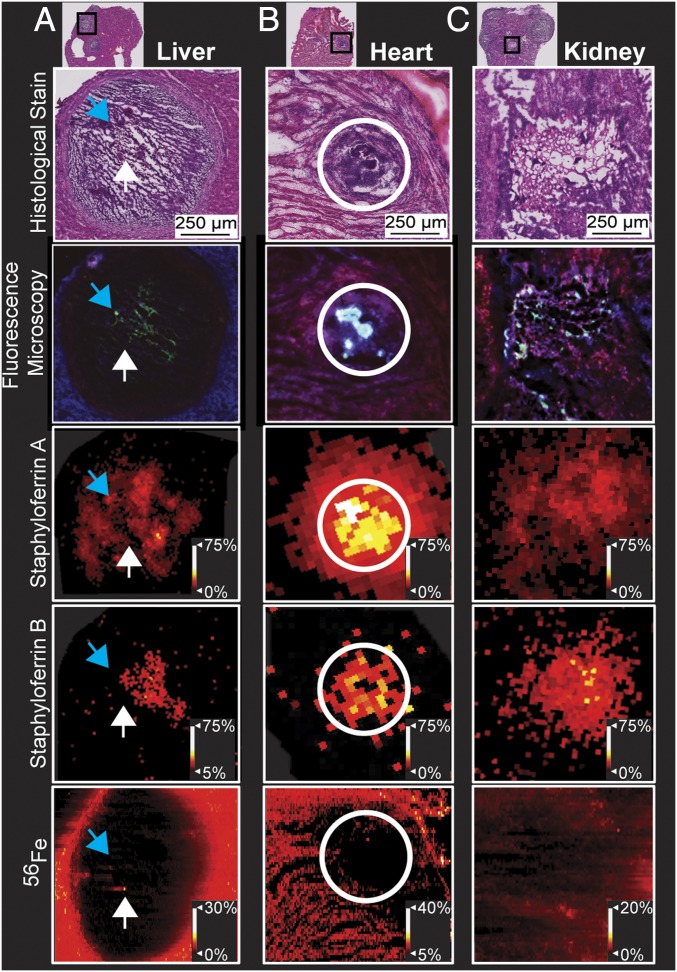
Multimodal imaging of 10-DPI *S. aureus P*_*isdA*_*gfp* infection characterizes utilization of SA and SB across tissue types. (*A*) Siderophore distributions localize to regions of staphylococcal Fe starvation (blue arrows). Fe distributions colocalize to areas that lack GFP signal in the fluorescent micrograph (white arrows). (*B*) Siderophore distributions in the heart expand outside of the abscess. (*C*) Heterogeneity in siderophore and Fe distributions as well as Fe starvation can be observed from zooms of a single kidney abscess, similar to distributions within the liver.

These results provide insight into staphylococcal metal acquisition during infection and emphasize the capabilities of IMS to investigate host−microbe interactions. While it is accepted that siderophores play a role in pathogenesis, it is less clear why bacteria produce multiple distinct siderophores. In addition to Fe, glucose represses SA production, and heme affects SB production (7, 22, 23). Furthermore, SA and SB differentially impact infection outcomes in murine models (5, 7, 21, 22). These results suggest a niche-specific role for each siderophore, rather than functional redundancy. Differential distributions of these siderophores may be explained by molecular heterogeneity within the abscess. Use of spatial molecular technologies such as MALDI IMS reveals siderophore distributions in tissue, and, when combined with multimodality integration, enables an unprecedented view of the struggle for metal between host and pathogen. The ability to image bacterial metabolites within tissue has the potential to be broadly applicable to infection biology, microbiome studies, and clinical microbiology.
